# Serotype Distribution and Characteristics of the Minimum Inhibitory Concentrations of *Streptococcus pneumoniae* Isolated from Pediatric Patients in Kunming, China

**DOI:** 10.1007/s00284-021-02365-4

**Published:** 2021-02-18

**Authors:** Mingbiao Ma, Mei Yuan, Ming Li, Xiaojuan Li, Hailin Huang, Haiping Wang, Jue Li, Tingyi Du, Rongwei Huang

**Affiliations:** 1 Department of Clinical Laboratory, Kunming Children’s Hospital, Kunming, 650500 China; 2 Yunnan Key Laboratory of Children’s Major Disease Research, Kunming, 650500 China; 3 Department of Respiratory Medicine, Kunming Children’s Hospital, Kunming, 650500 China; 4 Department of Respiratory and Critical Care, Kunming Children’s Hospital, Kunming, 650500 China

## Abstract

*Streptococcus pneumoniae* (*S. pneumoniae*) is the main conditional pathogen of acute respiratory infection in infants, children, and older adults worldwide. It was great significant to identify the epidemic characteristics of serotypes and antibiotic susceptibility for the prevention and treatment of *S. pneumoniae* diseases. This research assessed the serotype distribution and the minimum inhibitory concentrations (MICs) of *S. pneumoniae* isolated from pediatric patients to provide information on the epidemiology and antibiotic resistance of *S. pneumoniae* in Kunming, China. A total of 140 *S. pneumoniae* isolates were collected from pediatric patients at Kunming Children’s Hospital from January 2016 to October 2017. Serotype identification was done by Quellung reaction and multiplex polymerase chain reaction. MICs were determined by *E*-test. 140 isolates distributed in 13 types of serotypes. The top-three prevalent serotypes were 19F, 19A, and 6B. The immunization coverage rate of 13-valent pneumococcal conjugate vaccine (PCV) was relatively higher and should be introduced into the vaccination program in the region. MIC_50_ of penicillin, ceftriaxone, and levofloxacin was 1 μg/mL. MIC_50_ for meropenem and vancomycin was 0.38 μg/mL. MIC_90_ of penicillin, ceftriaxone, and levofloxacin was 1.5 μg/mL and that of meropenem and vancomycin was 0.5 μg/mL. The MIC_90_ of erythromycin was > 256 μg/mL. In summary, *S. pneumoniae* had low resistance rates to penicillin, ceftriaxone, levofloxacin, vancomycin, and meropenem, and these antibiotics could be the first-line agents for children with pneumococcal infections in Kunming.

## Introduction

*Streptococcus pneumoniae* (*S. pneumoniae*) is a Gram-positive bacteria. It is the most common pathogenic bacterial species that causes community-acquired infection in children, and can cause meningitis, otitis media, sinusitis, bacteremia, and pneumonia. It can even endanger life in severe cases [1]. According to a World Health Organization (WHO) report, there were about 700,000 to 1,000,000 children worldwide die from pneumococcal infections every year, and most are children under 5 years of age [2, 3]. The population of China is the largest in the world, and about 350,000 children aged < 5 years died from pneumococcal infections each year from 2010 to 2015 [4]. This figure accounted for 10% of children who died worldwide from pneumococcal infections. Therefore, pneumococcal infections in children have become an important public-health issue all over the world and aroused the great attention of medical circles.

At the microscope level, there is a double-spherical arrangement of capsules, which are the main virulence factors of *S. pneumoniae*. Depending on the structure and composition of the capsular polysaccharide, *S. pneumoniae* can be divided into 46 serogroups and 90 serotypes at present. About 20 serotypes are associated with common *S. pneumoniae*-related diseases [5].

However, to reduce the disease burden of pneumococcal infections, two types of multivalent vaccines, a pneumococcal polysaccharide vaccine (PPV) and pneumococcal conjugate vaccines (PCVs), have been developed and proven to be an effective method for preventing pneumococcal infections worldwide. The 23-valent pneumococcal polysaccharide vaccine (PPV23) can target 23 pneumococcal serotypes (1, 2, 3, 4, 5, 6B, 7F, 8, 9N, 9V, 10A, 11A, 12F, 14, 15B, 17F, 18C, 19A, 19F, 20, 22F, 23F, and 33F). Three types of PCVs are in use. 7-valent pneumococcal conjugate vaccine (PCV7) can target seven pneumococcal serotypes (4, 6B, 9V, 14, 18C, 19F, and 23F). PCV11 contains additional serotypes 1, 3, 5, and 7F, and PCV13 includes extra 6A and 19A. PPV23 has a higher coverage rate of serotypes compared with any PCVs, but whether it produces protective antibodies is dependent upon stimulating B cells. PPV23 cannot produce a strong immune protective effect for infants and children aged < 2 years. PCVs can activate T cells to provide sufficient immunologic help to produce antibodies and to stimulate immunological memory. PCVs are recommended for infants and young children with relatively immature B cells.

Currently, PPV23 and PCV7 are available commercially in China. However, several studies have found that serotypes are distributed differently in different periods, geographic areas, and populations [6]. Whether existing multivalent pneumococcal vaccines are suitable for children is dependent upon the serotype distribution of clinical pneumococcal isolates in a particular region.

Antibiotics are first-line treatment against pneumococcal infections, especially β-1Lactam antibiotics such as penicillin and ceftriaxone. However, with the widespread use of antibiotics in recent years, *S. pneumoniae* has become resistant to various antimicrobial drugs. In particular, the emergence of penicillin-resistant *S. pneumoniae* (PRSP) has become a big challenge for clinical treatment [7, 8].

Kunming, the capital city of Yunnan Province, is an important gateway to South Asia. This Chinese city has a population of seven million people. However, the development of economy and health care here has lagged behind that of other cities in China. Most of epidemiologic data of *S. pneumoniae* mainly focused on several developed cities including Beijing, Shanghai, and Guangzhou. The children here are rarely vaccinated with pneumococcal vaccines. Epidemiology and drug-resistance information for *S. pneumoniae* have been not yet available for Yunnan province.

We investigated, for the first time, the essential information about serotypes distribution and antibiotic resistance of *S. pneumoniae* in Kunming. This information is very important for *S. pneumoniae* infections in children in this region, especially for the introduction and policies of vaccination.

## Materials and Methods

### Collection of Bacterial Isolates

From January 2016 to October 2017, a total of 140 *S. pneumoniae* isolates were collected from pediatric patients at the Kunming Children's Hospital. These bacterial isolates were obtained from sputum, blood, eye secretions, broncho-alveolar lavage fluid (BALF), and middle-ear fluid. We inoculated the specimens onto agar plates containing 5% sheep red blood cells (Autobio Diagnostics, Zhengzhou, China). We cultured them in an atmosphere of 5% CO_2_ for 18–24 h at 37 °C. We found the suspected colonies to be concave and accompanied by *α*-hemolysis. Further confirmation was achieved by the presence of diplococci on Gram stain under the microscope views, test (optochin sensitivity, the bile susceptibility), and by use of an automatic bacterial identification system (VITEK2-compact, bioMerieux, Marcy-l'Étoile, France). These isolates were stored at −80 °C in frozen tubes containing equine serum (Microbank™, Pro Lab Diagnostics, Richmond Hill, ON, USA).

### Detection of Serotypes

The serotypes/groups of all 140 strains were identified first with a Pneumotest-Latex kit (Statens Serum Institut, Copenhagen, Denmark) comprising 14 latex reagents pools (A to I and P to T). The kit could identify the 23 vaccine serotypes at the type/group level by all 14 pools using the “chessboard” identification system. Type-specific antisera were used for full serotypes of serogroups 19, 6, and 15. Strains that could not be serotyped by the Quellung reaction were finally confirmed using multiplex polymerase chain reaction (PCR) targeting the cps locus using primers designed previously [9]. PCR was done in a 50μL PCR mixture containing 25μL of 2× Multiplex PCR Buffer, 0.5μL of 0.2 μM of each primer, 0.25μL of Multiplex PCR Enzyme Mix, 19.75μL of dH_2_O, and 1μL of DNA template extracted from isolates (TaKaRa Biotechnology, Shiga, Japan). PCR products were visualized by electrophoresis on 1% agarose gel. The immunization coverage rates of PCV7, PCV13, and PPV23 were estimated by calculating the percentages of strains targeted by each vaccine in all of those isolates.

### Antimicrobial Susceptibility Test

*E*-test and Mueller Hinton agar plates containing 5% red blood cells from sheep (Autobio Diagnostics) were used to assess the minimum inhibitory concentrations (MICs) of 140 *S. pneumoniae* isolates for susceptibility to penicillin, ceftriaxone, meropenem, levofloxacin, vancomycin, and erythromycin. The results of the susceptibility testing to antibiotics were determined according to the guidelines provided by the Clinical and Laboratory Standards Institute (CLSI) 2018 [10]. For the susceptibility breakpoint of penicillin, MIC ≤ 2 μg/mL was defined as “susceptible,” 4 μg/mL was “intermediate,” and MIC ≥ 8 μg/mL was “resistant.” In addition, ATCC49619 was used as the quality-control strain to ensure the accuracy of results in each test. MIC_50_ and MIC_90_ represented the antibiotic concentrations at which 50% and 90% of the bacterial isolates could be inhibited, respectively.

### Statistical Analysis

The data of the antimicrobial susceptibility were analyzed using WHONET 5.6 software (WHO, Geneva, Switzerland).

## Results

### Information of Pediatric Patients and Isolates

*Streptococcus pneumoniae* isolates were collected from 140 patients aged from 4 months to 9 years. The proportion of boys accounted for 58.57% (82/140) and that of girls was 41.43% (58/140). Sputum was the most common specimen source, accounting for 94.29% of samples (132 isolates), followed by eye secretions (4 isolates), middle-ear fluid (2 isolates), blood (1 isolate), and BALF (1 isolate).

### Serotype Distribution and Immunization Coverage Rates of Pneumococcal Vaccines

Of all 140 isolates, 139 isolates were identified and distributed in 13 types of serotypes. One isolate could not be identified. The serotype distribution of all 140 *S. pneumoniae* isolates in different age groups is shown in Table 1. In children aged < 2 years and children aged ≥ 2 years, the most prevalent serotype was 19F (28.0% and 39.2%, respectively), followed by 19A (22.4% and 17.6%, respectively) and 6B (14.6% and 13.7%, respectively). Rare serotypes such as 6D, 7C, and 10A appeared mainly in children aged < 2 years.

**Table 1:** Serotype distribution of 140 *S. pneumoniae* isolates in different age groups

Serotype	All children *n* (%)	Children aged < 2 years *n* (%)	Children aged ≥ 2 years *n* (%)
19F	45 (32.1%)	25 (28.0%)	20 (39.2%)
19A	29 (20.7%)	20 (22.4%)	9 (17.6%)
6B	20 (14.2%)	13 (14.6%)	7 (13.7%)
15B/C	13 (9.2%)	7 (7.8%)	6 (11.7%)
6A	8 (5.7%)	4 (4.4%)	4 (7.8%)
23F	8 (5.7%)	5 (5.6%)	3 (5.8%)
15A	6 (4.2%)	5 (5.6%)	1 (1.9%)
14	3 (2.1%)	3 (3.3%)	0
6C	3 (2.1%)	3 (3.3%)	0
6D	1 (0.7%)	0	1 (1.9%)
7C	1 (0.7%)	1 (1.1%)	0
20	1 (0.7%)	1 (1.1%)	0
10A	1 (0.7%)	1 (1.1%)	0
Untyped	1 (0.7%)	1 (1.1%)	0
Total	140	89	51

**Fig. 1:**
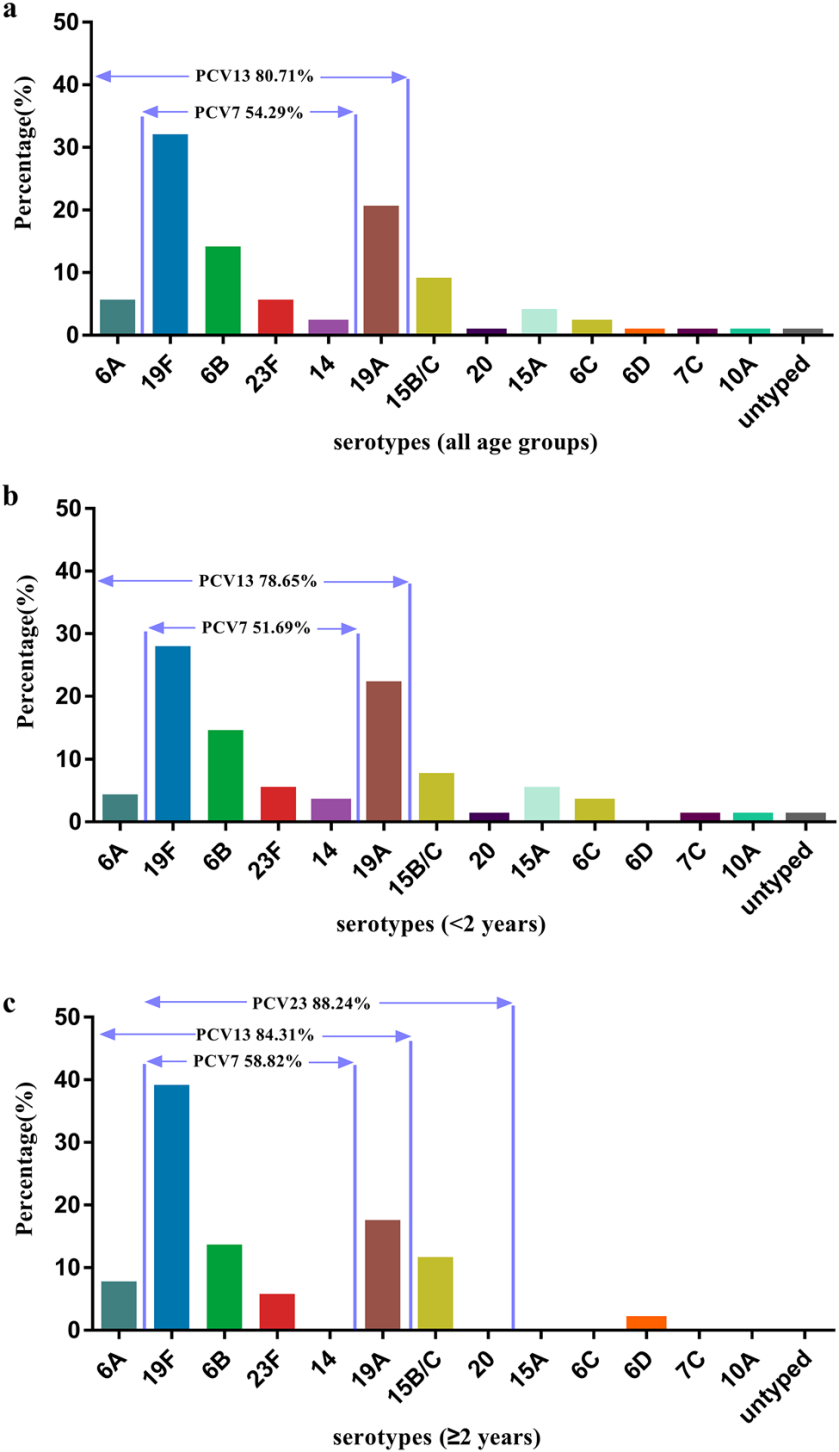
Serotypes and immunization coverage rates of **a** PCV7 and PCV13 in 140 *S. pneumoniae* isolates, **b** PCV7 and PCV13 among children aged < 2 years, and **c** PCV7, PCV13, and PPV23 among children aged ≥ 2 years

Of all 140 isolates, the immunization coverage rate of PCV7 and PCV13 was 54.29% (76/140) and 80.71% (114/140), respectively. In addition, the immunization coverage rate of PCV7 and PCV13 in children aged < 2 years (51.69% and 78.65%, respectively) was lower compared that that of children aged ≥ 2 years (58.82% and 84.31%, respectively). The immunization coverage rate of PPV23 in children aged ≥ 2 years was higher than that of any PCV (Fig. 1).

### Antimicrobial Susceptibility Test

**Fig. 2:**
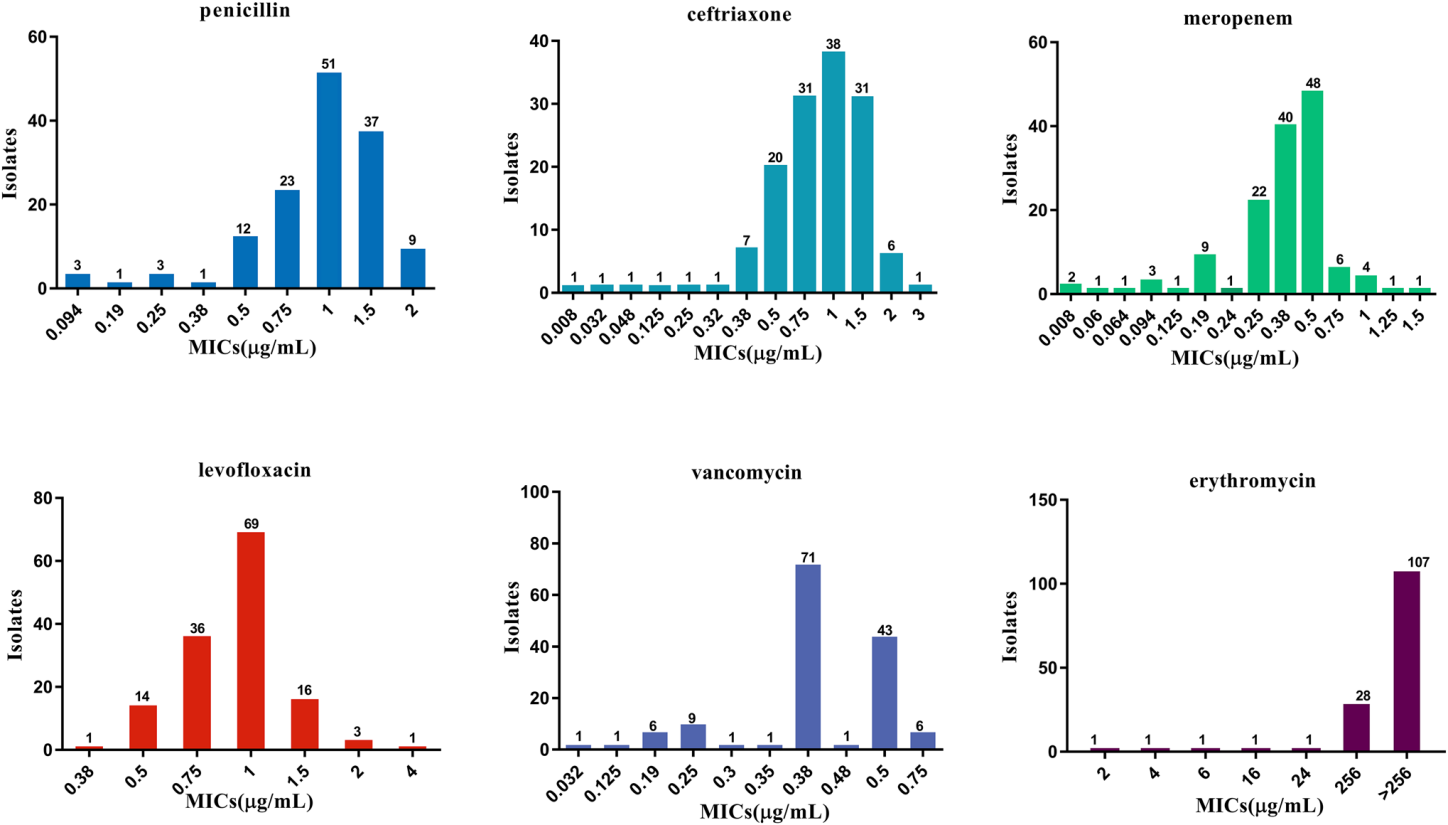
Distribution of MICs of 140 *S. pneumoniae* isolates against six common antibiotics

The MIC_50_ and MIC_90_ of the 140 *S. pneumoniae* isolates against to six common antibiotics are shown in Table 2. All results of ATCC49619 were within the reference range. The MIC_50_ of meropenem and vancomycin was 0.38 μg/mL and that of penicillin, ceftriaxone, and levofloxacin was 1 μg/mL. The MIC_90_ of penicillin, ceftriaxone, and levofloxacin was 1.5 μg/mL, and that of meropenem and vancomycin was 0.5 μg/mL. The MIC_50_ and MIC_90_ values of erythromycin were > 256 μg/mL. The distributions of MICs for the 140 *S. pneumoniae* isolates against to six common antibiotics are shown in Fig. 2. According to the CLSI 2018, no isolate resistant to penicillin, ceftriaxone, levofloxacin, or vancomycin was found. The resistance rate of 140 isolates to meropenem and erythromycin was 5% and 100%, respectively.

**Table 2:** The MIC_50_ and MIC_90_ of 140 *S. pneumoniae* isolates and distribution of MICs of isolates and ATCC49619 against to six common antibiotics

Antibiotic	MIC_50_ (μg/mL)	MIC_90_ (μg/mL)	MIC range of isolates (μg/mL)	MIC range of ATCC49619 (μg/mL)
Penicillin	1	1.5	0.094–2	0.25–0.38
Ceftriaxone	1	1.5	0.008–3	0.032–0.064
Meropenem	0.38	0.5	0.008–1.5	0.032–0.064
Levofloxacin	1	1.5	0.38–4	0.5
Vancomycin	0.38	0.5	0.032–0.75	0.19–0.38
Erythromycin	> 256	> 256	2– > 256	0.094

**Fig. 3:**
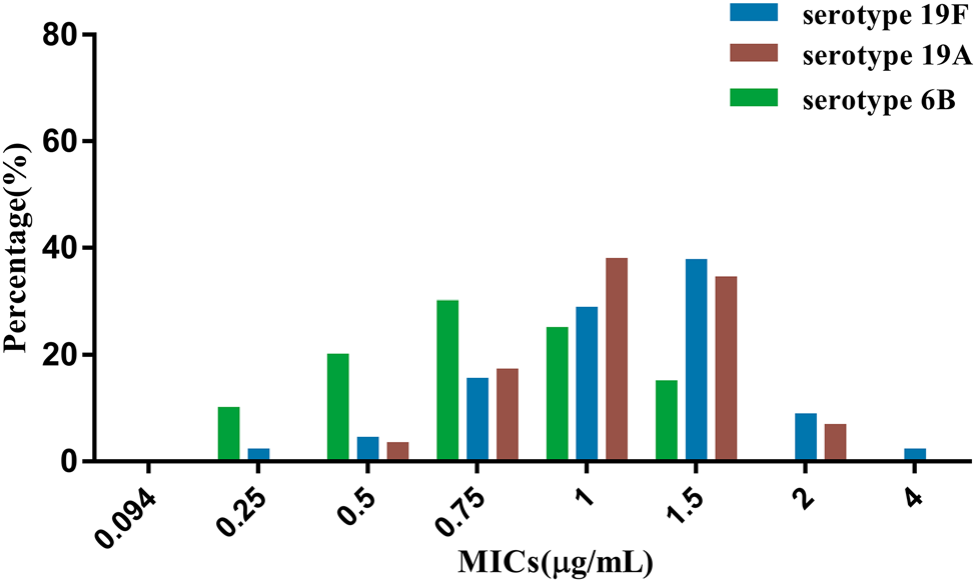
Distribution of MICs of serotypes19F, 19A and 6B against penicillin

Furthermore, we analyzed the MICs of predominant serotypes (19F, 19A, and 6B) against penicillin. The distribution of MICs (in μg/mL) of serotypes 19F, 19A, and 6B was concentrated at 1.5, 1.0, and 0.75, respectively (Fig. 3). At this time, we calculated the MIC_50_ and MIC_90_ of penicillin and found that the MIC_50_ and MIC_90_ of serotype 19A were 1 and 1.5 μg/mL, respectively, which was similar to the results of the 140 *S. pneumoniae* isolates. However, the MIC_50_ of serotypes 19F and 6B for penicillin both were 0.75 μg/mL and less than that of the 140 *S. pneumoniae* isolates.

## Discussion

*Streptococcus pneumoniae* is the main conditional pathogen of acute respiratory infection in infants, children, and senior citizens worldwide. It also can cause large-scale epidemics and seriously endanger human health under certain environment [11]. In recent years, the resistance rates of *S. pneumoniae* have been increasing year by year, which have been related to the application of antibiotics in some areas. Hausdorff and colleagues revealed significant differences in the serotypes and resistance rates of *S. pneumoniae* in different regions [6]. Here, we showed, for the first time, the serotype distribution and the MICs of *S. pneumoniae* to provide information on the epidemiology and antibiotic resistance of *S. pneumoniae* in Kunming.

Identification of the epidemic characteristics of serotypes is extremely important for the prevention and treatment of *S. pneumoniae*-related diseases. To understand the serotype distribution of *S. pneumoniae*, surveillance has been carried out in various regions and countries. One systematic evaluation showed that the most common serotypes worldwide to be 1, 5, 6A, 6B, 14, 19F, and 23F [12]. In Canada, the most common serotypes were 19A, 7F, 3, and 22F. Serotypes 19A, 5, 7F, and 3 were predominant in Spain [13]. However, the dominant serotypes in China are 19F, 19A, 23F, 14, and 6B [14]. Hence, the serotypes of *S. pneumoniae* may vary with ethnicity and region. From the serotype distribution of 140 *S. pneumoniae* isolates in the present study, 14 serotypes of *S. pneumoniae* were carried by pediatric patients in Kunming. The distribution was relatively concentrated, mainly serotypes 19F, 19A, and 6B. These data are in accordance with the main epidemic serotypes of children documented in Shanghai, Suzhou, and Chongqing [15–17] but different for those from Beijing (19F, 14, 23F, 6B, and 19A) and Chengdu (19F, 6, 14, 23F, and 15B/C) [18, 19]. Besides, Shen and colleagues found that 19F, 6A/B, 19A, and 23 were frequent serotypes in Zunyi (a city near to Kunming) [20]. Such results may suggest that the difference in serotype distribution between areas close to each other is not obvious.

Serotyping of *S. pneumoniae* and evaluating the application of pneumococcal vaccines are important steps to prevent and treat pneumococcal infections and to understand their epidemic characteristics. PCV7 and PPV23 have been approved for marketing in China recently. Infants aged 3 to 23 months and children aged 24 to 59 months not vaccinated with PCV7 are recommended to be vaccinated with PCV7, whereas children ≥ 2 years of age and adults can be vaccinated with PPV23. We showed that the immunization rate of PCV7 in children aged < 2 years and that of PPV23 in children aged ≥ 2 years was 51.69% and 88.24%, respectively. These data suggested that promotion of vaccination using PCV7 and PPV23 had preventive importance in China.

However, with the gradual promotion of a vaccine, some non-vaccine serotypes can increase in the proportion of isolates (“serotype replacement”) [21]. In the present study, the increase in non-vaccine serotype 19A was 20.7%. Because of targeting of serotype 19A, the immunization coverage rate of PCV13 increased and accounted for 80.71%. Based on the serotype distribution of *S. pneumoniae* in our study, we believe that PCV13 should be introduced into the vaccination program in Kunming, and it may protect a greater proportion of the population from pneumococcal infections.

The resistance rate of penicillin in China is slightly lower than that in neighboring countries and regions, and the antibiotic resistance rates of *S. pneumoniae* among children vary greatly in different regions [22]. According to the results of CHINET in 2017, the resistance rates of penicillin, ceftriaxone, levofloxacin, vancomycin, and erythromycin in children and newborns were 1.6%, 13.2%, 0.7%, 0% and 96.8%, respectively, across China [23]. We showed that the resistance rates of penicillin, ceftriaxone, and levofloxacin in Kunming were lower than that in China. Six isolates were resistant to meropenem. The resistance rate of meropenem was lower than that reported by Jing et al. [17]. The reasons for this difference may be (i) because we used *E*-tests for drug- susceptibility testing, which is more likely to avoid false-positive resistance values than that using routine clinical methods and (ii) related to the region where is accustomed to use the ceftriaxone or meropenem rather than penicillin to treat pneumococcal infections.

Since the first discovery of PRSP in Australia in 1967, the resistance of *S. pneumoniae* to antibiotics such as *β*-1Lactams, macrolides, and tetracycline has been reported widely, and the resistance rate of penicillin is related to the geographic distribution [8, 24, 25]. Although *S. pneumoniae* has 90 serotypes, the serotype distribution of PRSP is relatively concentrated. In the United States, 96% of penicillin-resistant serotypes of *S. pneumoniae* are 6B, 14, 23F, 9V, 19A, and 19F. In Canada, 23F, 19A, 6B, and 9V are the main serotypes resistant to penicillin, and 14.82% of PRSP belong to serotypes 23F, 19A, 14, 6B, and 9V in France [6]. However, we did not find a PRSP isolate, which may be because penicillin is not often used as the first-line agent against pneumococcal infections in Kunming.

Ascertaining the distribution of MICs of antibiotics is significant conducive to surveying the changing trends of resistance rates in an area and guiding clinical antibiotic use. In our study, the distribution of MICs of penicillin was mainly 0.5–2 μg/mL, and that of MIC_50_ and MIC_90_ was 1 μg/mL and 1.5 μg/mL, respectively. These data indicate that the sensitivity of penicillin to *S. pneumoniae* in Kunming was high, and that penicillin could be used for treatment of *S. pneumoniae* infections in Kunming. However, the characteristics of MICs in different serotypes showed differences. The distributions of MICs of serotypes 19F, 19A, and 6B was concentrated at 1.5, 1, and 0.75 μg/mL, respectively, in the present study. The MIC_50_ in serotypes 19F and 6B was 0.75 μg/mL and was < 1 μg/mL in 19A. Although this change had no significant effect on qualitative determination of penicillin in our study, further investigation of the distribution of MICs of serotypes is necessary for long-term monitoring.

## Conclusions

We found that 19F, 19A, and 6B were the most common serotypes of *S. pneumoniae* isolated from pediatric patients in Kunming. PCV13 was more useful than PCV7 in the region to prevent the spread of pneumococcal infections. *S. pneumoniae* had low resistance rates to penicillin, ceftriaxone, levofloxacin, vancomycin, and meropenem, and these antibiotics could be the first-line treatment for children with pneumococcal infections in this region. Moreover, larger sample size and longer monitoring should also be included in future studies to confirm our results and provide more detailed information on the epidemiology and antibiotic resistance of *S. pneumoniae* for the region.
